# Environmentally-induced *mdig* contributes to the severity of COVID-19 through fostering expression of SARS-CoV-2 receptor NRPs and glycan metabolism

**DOI:** 10.7150/thno.62138

**Published:** 2021-07-06

**Authors:** Qian Zhang, Priya Wadgaonkar, Liping Xu, Chitra Thakur, Yao Fu, Zhuoyue Bi, Yiran Qiu, Bandar Almutairy, Wenxuan Zhang, Paul Stemmer, Fei Chen

**Affiliations:** 1 Department of Pharmaceutical Sciences, Eugene Applebaum College of Pharmacy and Health Sciences, Wayne State University, 259 Mack Avenue, Detroit, MI 48201, USA.; 2 Stony Brook Cancer Center and Department of Pathology, Stony Brook University, Lauterbur Drive, Stony Brook, NY 11794, USA.; 3 Institute of Environmental Health Sciences, School of Medicine, Wayne State University, 540 E. Canfield St, Detroit, MI 48201, USA.

**Keywords:** *mdig*, COVID-19, NRP1, NRP2, Glycosylation

## Abstract

The novel β-coronavirus, SARS-CoV-2, the causative agent of coronavirus disease 2019 (COVID-19), has infected more than 177 million people and resulted in 3.84 million death worldwide. Recent epidemiological studies suggested that some environmental factors, such as air pollution, might be the important contributors to the mortality of COVID-19. However, how environmental exposure enhances the severity of COVID-19 remains to be fully understood. In the present report, we provided evidence showing that *mdig*, a previously reported environmentally-induced oncogene that antagonizes repressive trimethylation of histone proteins, is an important regulator for SARS-CoV-2 receptors neuropilin-1 (NRP1) and NRP2, cathepsins, glycan metabolism and inflammation, key determinants for viral infection and cytokine storm of the patients. Depletion of *mdig* in bronchial epithelial cells by CRISPR-Cas-9 gene editing resulted in a decreased expression of NRP1, NRP2, cathepsins, and genes involved in protein glycosylation and inflammation, largely due to a substantial enrichment of lysine 9 and/or lysine 27 trimethylation of histone H3 (H3K9me3/H3K27me3) on these genes as determined by ChIP-seq. Meanwhile, we also validated that environmental factor arsenic is able to induce *mdig*, NRP1 and NRP2, and genetic disruption of *mdig* lowered expression of NRP1 and NRP2. Furthermore, *mdig* may coordinate with the Neanderthal variants linked to an elevated mortality of COVID-19. These data, thus, suggest that *mdig* is a key mediator for the severity of COVID-19 in response to environmental exposure and targeting *mdig* may be the one of the effective strategies in ameliorating the symptom and reducing the mortality of COVID-19.

## Introduction

The global outbreak of COVID-19, caused by the newly identified SARS-CoV-2 virus, is having a devastating impact on both public health and social economics. According to the World Health Organization (WHO), as of June 20, 2021, there had been more than 177 million confirmed cases of COVID-19 worldwide, leading to almost 3.84 million deaths. The SARS-CoV-2 virus has a large number of trimeric spikes (S) with many unique salient features on its surface. Each S protein has two subdomains, S1 and S2. Upon direct binding of S1 to the ACE2 on the surface of the host cells, membrane-associated proteases of the host cells, including furin, TMPRSS2, and others, cleave S proteins at the RRAR/S motif located at the S1-S2 boundary, or the motif of PSKR/S, the so-called S2' site, in S2 region, followed by S2-mediated membrane fusion 1-3. In addition to ACE2, the C-terminal RRAR motif of the S1 protein from the cleaved S protein may bind to cell surface neuropilin-1 (NRP1) or NRP2 for sufficient viral entry into the host cells 4-6. After membrane fusion, the virus is shuttled in the cytoplasm through the endosomes and lysosomes, during which several cathepsin family proteases cleave S proteins further and other viral proteins, leading to the release of the RNA genome of SARS-CoV-2 into the cytoplasm 2. Indeed, some inhibitors targeting endosome, lysosome or cathepsin, such as ammonia chloride, bafilomycin A and teicoplanin, had been shown to be effective in blocking viral entry in cell-based experiments 2, 7. The viral RNA genome can hijack the protein translational machinery of the host cells for the expression of viral polyproteins that can be processed to generate replicase, proteases, transcriptases, and other viral structural proteins for assembly of the new virion. Some of these newly generated viral proteins can antagonize the anti-viral responses of the interferon signaling, whereas others are highly capable of activating the inflammasome for cytokine storm and pyroptosis of the host cells in lung and other organs, leading to an increased likelihood of fatality of the COVID-19 patients 8.

Both S protein and ACE2 are known to be extensively glycosylated through covalently linked oligosaccharides (glycans) 9. It is believed that glycosylation may enhance the infectivity of the virus. Many glycoconjugates on the surface of the host cells are also modified by sialic acid, a specific form of glycosylation—sialylation, which may facilitate membrane fusion of the virus and cells. It is also very likely that alteration in glycan profile on ACE2 and cell membrane may determine the inter-individual variation on the outcomes of viral infection 10.

Human lung is the first line of attack by the SARS-CoV-2 virus. The fatality of COVID-19 has been largely attributed to the massive alveolar damage and progressive respiratory failure resulted from cellular fibromyxoid exudates, formation of hyaline (hyaluronan) membrane, pulmonary oedema, inflammation, and interstitial fibrosis 11-13. Most environmental risk factors, such as air pollution and PM2.5, can induce chronic inflammation and make the lung more vulnerable to viral attacks. Indeed, a recent nationwide epidemiological study in the US had shown that an increase of only 1 μg/m^3^ in PM2.5 is associated with an 8% increase in the COVID-19 death rate, clearly indicating that environmental factors may contribute to the severity of the disease outcomes among individuals with COVID-19 14. This notion is complementarily supported by the fact that the areas, such as Lombardy, Emilia-Romanga, Piemonte, and Veneto of Italy, and Madrid of Spain, with worse air quality due to higher level of nitrogen dioxide (NO2), are the hardest-hit areas with deaths from COVID-19 15. In addition, exposure to some industrial chemicals, such as the perfluorinated alkylate substances (PFASs), may exacerbate the severity of COVID-19 16.

The mineral dust-induced gene *mdig* was first identified in alveolar macrophages from people with chronic lung diseases associated with occupational exposure 17, and additional studies concluded that *mdig* can be induced by a number of environmental hazards, such as silica particles, arsenic, tobacco smoke, and PM2.5 17. This gene was independently discovered as myc-induced nuclear antigen (mina53) and nucleolar protein NO52, respectively. Functional tests suggested that *mdig* may have hydroxylase activity on ribosomal protein L27a, and accordingly, an alternative name, *riox2*, was given 17. The *mdig* protein contains a conserved JmjC domain that was considered as a signature motif of the histone demethylase family members. In human bronchial epithelial cells, lung cancer cell line A549, and breast cancer cell line MDA-MB-231 cells, knockout of *mdig* gene by CRISPR-Cas9 gene-editing, resulted in a pronounced enrichment of histone H3 lysine9 trimethylation (H3K9me3) as well as H3K27me3 and H4K20me3 in ChIP-seq analysis, esp. in the gene loci encoding proteins in inflammation and fibrosis, such H19, TGFβ signaling, collagens, and cell adhesion molecules 18. Data from mice with heterozygotic deletion of *mdig* gene indicated that *mdig* is a master regulator of inflammation and tissue fibrosis 18, 19. In the present report, we further demonstrated that *mdig* may exacerbate the severity of COVID-19 in response to environmental exposure.

## Results

### Depletion of *mdig* prevents cleavage of SARS-CoV-2 spike protein

**Figure 1:**
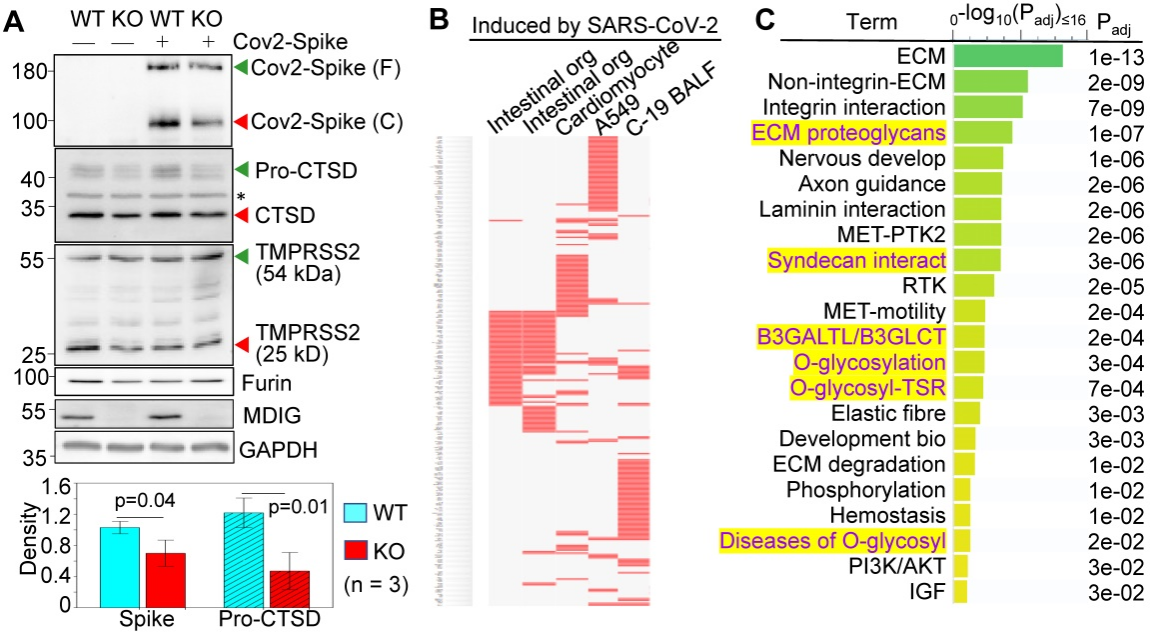
** Knockout of *mdig* diminishes the expression of genes for SARS-CoV-2 infectivity. A.** Western blotting shows decreased cleavage of the transfected SARS-CoV-2 S protein and the expression of CTSD in *mdig* KO BEAS-2B cells. Data are representatives of at least three independent experiments. Bottom panel shows ImageJ quantifications of the cleaved SARS-CoV-2 spike protein and pro-CTSD of three independent experiments of the WT and KO cells. The data were calibrated by the density of GAPDH or histone H3. **B.** The down-regulated genes in the *mdig* KO cells as determined by RNA-seq are highly represented for those gene induced by SARS-CoV-2 in intestinal organoids (GSE149312), cardiomyocytes (GSE150392), A549 cells (GSE147507), and bronchoalveolar lavage fluid (BALF) of COVID-19 patients. **C.** Reactome pathway assay shows the down-regulated genes in *mdig* KO cells as determined by RNA-seq are mostly in the pathways of extracellular matrix (ECM) regulation and glycan metabolism (highlighted in yellow).

We had used non-cancerous bronchial epithelial cell line BEAS-2B to establish *mdig* knockout (KO) cells through CRISPR-Cas9 gene-editing, and used the cells subjected to gene editing but without *mdig* depletion as wild type (WT) cells 18. By transfection of the WT and KO cells with an expression vector for the full-length spike (S) protein of SARS-CoV-2, a detectable decrease in S protein cleavage was noted in the *mdig* KO cells (Fig. 1A, top panel). The molecular weight (MW) of the unprocessed full-length S protein is around 200 kDa (green arrow head), and the cleaved product is about 100-110 kDa (red arrow head). To determine whether the decreased cleavage of S protein in *mdig* KO cells is a result of the diminished expression of proteases responsible for S protein cleavage, we compared the protein levels of cathepsin D (CTSD), transmembrane serine protease 2 (TMPRSS2), and furin between WT and *mdig* KO cells. There is no significant difference in the levels of TMPRSS2 and furin between WT and KO cells in three independent experiments. However, a substantial decrease of CTSD, both the 46 kDa precursor and 28 kDa mature form, was observed in the *mdig* KO cells (Fig. 1A).

To additionally investigate the effect of *mdig* knockout on the genes linked to SARS-CoV-2 infection and pathology, we performed gene expression profiling between WT and *mdig* KO cells by two independent RNA-seq analyses. The differentially expressed genes identified in both sets of RNA-seq data were then subjected to gene set enrichment analysis with the Enrichr web-based application. This analysis showed that the down-regulated genes in the *mdig* KO cells were over-presented in the gene sets induced by SARS-CoV-2 in intestinal organoids (GSE149312), cardiomyocytes (GSE150392), A549 cells (GSE147507), and bronchoalveolar lavage fluid (BALF) of COVID-19 patients (Fig. 1B), suggesting that *mdig* enhances expression of genes involved in either SARS-CoV-2 infection or the pathogenesis of COVID-19. To further understand the biological functions of the down-regulated genes identified in RNA-seq in *mdig* KO cells, we applied enrichment assay of the Reactome. In this analysis, we found that the top Reactome pathways of these down-regulated genes in *mdig* KO cells are related to organization, interaction and regulation of extracellular matrix (ECM) (Fig. 1C). Unexpectedly, six of the twenty-two top-ranked Reactome pathways are centered on protein glycosylation or glycan metabolism, indicating that in addition to ECM, *mdig* is critical for the post-translational modification of proteins by carbohydrate moieties.

### Involvement of *mdig* in antagonizing H3K9me3 and/or H3K27me3

**Figure 2:**
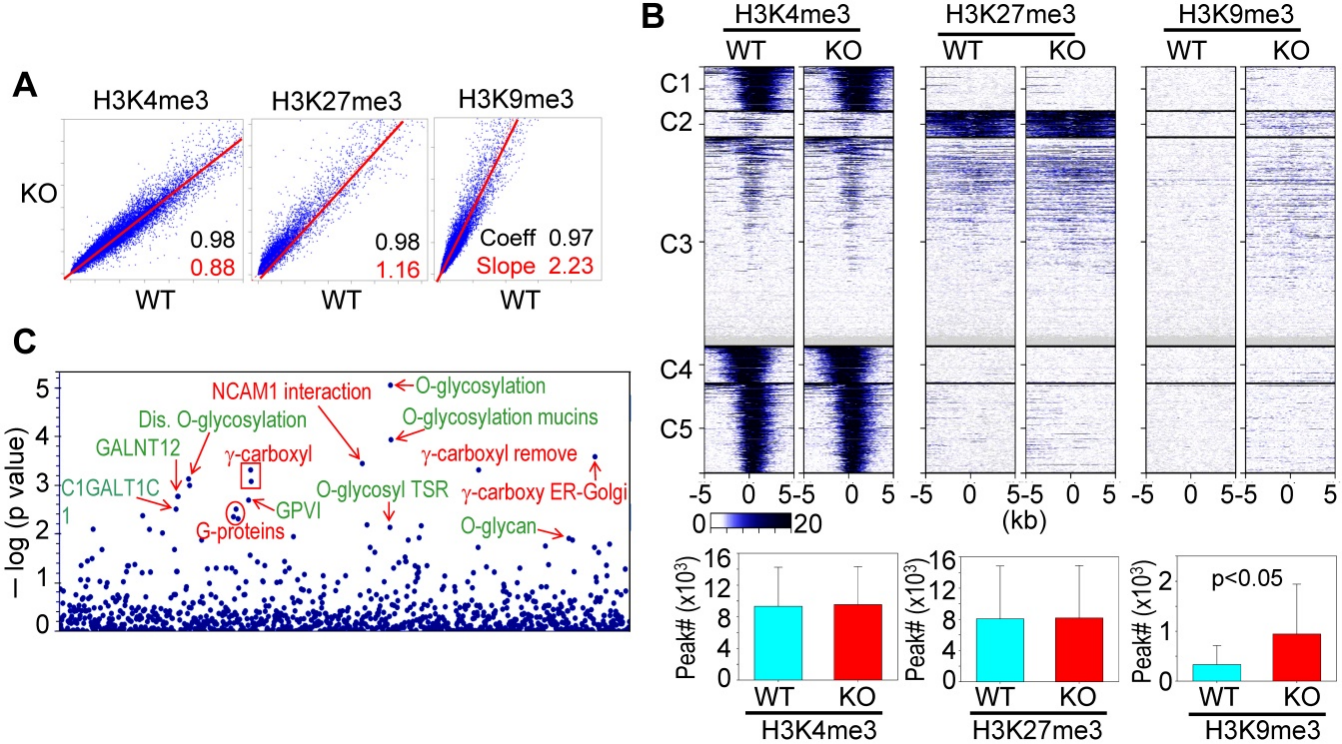
** ChIP-seq data revealed that *mdig* is antagonistic to H3K27me3 and H3K9me3. A.** Pairwise Pearson correlation analyses were made through plotting the tag numbers of H3K4me3, H3K9me3 and H3K27me3 in the genome in *mdig* KO cells against the corresponding tag numbers in WT cells. **B.** Clustered heatmaps of ChIP-seq for H3K4me3, H3K27me3 and H3K9me3 in the promoter regions of WT cells and *mdig* KO cells. Bar graphs at the bottom show the total numbers of the indicated peaks in all regions of the genome in WT and *mdig* KO cells. **C.** Reactome pathway analysis of the genes showed significantly enhanced enrichment of H3K9me3 in *mdig* KO cells. The top-ranked pathways in glycan metabolism are marked in green color.

A previous study showed that knockout of *mdig* in BEAS-2B cells caused significant enrichments of H3K9me3, H3K27me3, and H4K20me3 on the merged peak regions as determined by ChIP-seq 18. Re-analysis of the ChIP-seq data from the WT and *mdig* KO cells by pairwise Pearson correlation analyses, through plotting the total tag numbers of H3K4me3, H3K9me3 and H3K27me3 in *mdig* KO cells against the corresponding tag numbers in WT cells, revealed a significant enhancement of H3K9me3 and H3K27me3 in *mdig* KO cells (Fig. 2A). Clustered heatmaps as depicted in Fig. 2B suggested that the enrichment of H3K9me3 and H3K27me3 were drastically enhanced in clusters 2 and 3 of the *mdig* KO cells, while little to no changes in the enrichment of H3K9me3 and H3K27me3 were observed in clusters 1, 4 and 5. The enhanced H3K9me3 in *mdig* KO cells was also supported by the significantly increased number of H3K9me3 peaks on the genome in ChIP-seq. In WT cells, the normalized number of H3K9me3 peaks in all regions is 3308, whereas in KO cells, this number is 9433 (Fig. 2B, bottom panels). To determine which sets of genes are enriched with H3K9me3 in *mdig* KO cells, we conducted Reactome pathway assay for those H3K9me3-enriched genes. It is interesting to note that the top-ranked pathways are mostly associated with protein glycosylation and glycan regulation (Fig. 2C), which is highly correlated to the pathways of the down-regulated genes in RNA-seq of the *mdig* KO cells (Fig. 1C). These data, thus, provide an unequivocal evidence indicating that *mdig* fosters the expression of genes in glycosylation and glycan metabolism through antagonizing the repressive histone methylation marker H3K9me3 as well as H3K27me3.

### Receptors and proteases required for SARS-CoV-2 infection are regulatory targets of *mdig*

**Figure 3:**
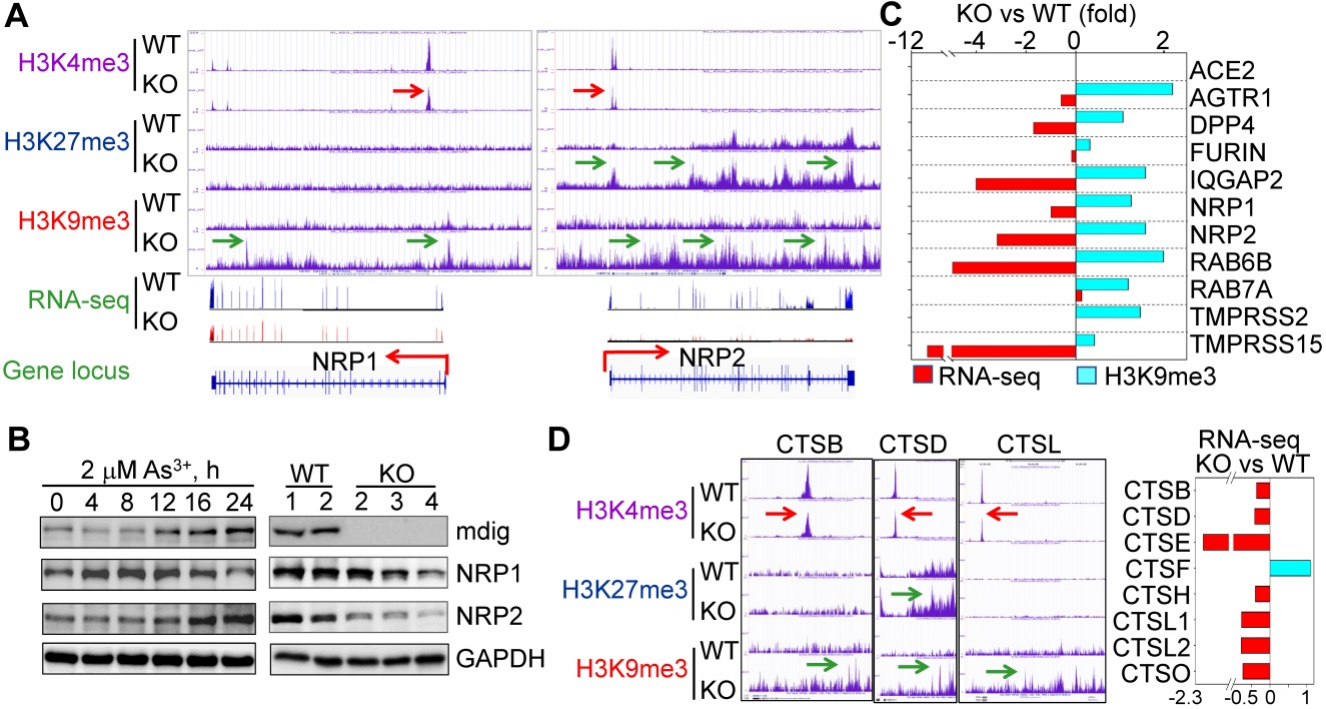
** Diminished expression of genes related to SARS-CoV-2 infectivity. A.** Down-regulation of S protein receptors NRP1 and NRP2 in *mdig* KO cells. Both ChIP-seq screenshot and RNA-seq spectrum for NRP1 and NRP2 were shown. Red arrows point to the reduced level of H3K4me3; green arrows denoted enhanced peaks of H3K9me3 and H3K27me3. **B.** Expression of NRP1 and NRP2 is *mdig* dependent. Left panels show time-dependent induction of *mdig*, NRP1 and NRP2 by As^3+^. Right panels show decreased level of NRP1 and NRP2 proteins in *mdig* KO cells as determined by Western blotting. **C.** The elevated enrichment of H3K9me3 as determined by ChIP-seq and reduced expression in RNA-seq of these indicated genes in *mdig* KO cells. **D.** Knockout of *mdig* elevated enrichment of H3K9me3 and/or H3K27me3 on the indicated cathepsin genes that also showed decreased enrichment of the active transcription marker, H3K4me3. Right panel: RNA-seq showed down-regulation of these indicated cathepsin genes in the *mdig* KO cells (except CTSF). Horizontal red and green arrows in Genome Browser screenshots indicate statistical differences, p < 0.05, of these enrichment peaks between WT and KO cells.

Considering the fact that knockout of *mdig* reduced cleavage of SARS-CoV-2 spike (S) protein (Fig.1A), we narrowed down our analysis of ChIP-seq and RNA-seq data to several key proteins important for S protein binding, intracellular vesicle trafficking and fusion. Upon close examination, we found that genes involved in key aspects of SARS-CoV-2 entry into the host cells, including NRP1, NRP2, AGTR1 (AT1), DPP4, TMPRSS15, RAB6B, IQGAP2, and others, are enriched with the repressive histone trimethylation marker, H3K9me3 or H3K27me3, along with a decreased expression of these genes as determined by RNA-seq, in *mdig* KO cells (Figs. 3A and 3C). NRP1 and NRP2 are transmembrane proteins serving as receptors for growth factors and semaphorins. Recent reports suggest that NRP1 or NRP2 can function as SARS-CoV-2 receptor through binding of the C-terminal RRAR motif of the furin-cleaved S protein, followed by internalization of the virus via micropinocytosis 4, 5. Knockout of *mdig* raised H3K9me3 in the up- and down-streams of NRP1 gene, and H3K27me3 and H3K9me3 (pointed by green arrows) in the gene body and down-stream of NRP2 gene, which are consistent with the diminished gene expression of NRP1 and NRP2 in *mdig* KO cells as determined by RNA-seq (Fig. 3A). Meanwhile, a drastic reduction of H3K4me3, an active transcription marker, was observed on the promoter region of both NRP1 and NRP2 genes in *mdig* KO cells (pointed by red arrows), which further enforces the transcriptional suppression of NRP1 and NRP2 by an increased level of H3K9me3 and/or H3K27me3 resulted from *mdig* deficiency. To investigate whether NRP1 and NRP2 expression is truly in a *mdig* dependent manner in cellular response to environmental factors, we next treated the cells with 2 μM arsenic (As^3+^), a well-established environmental carcinogen, for 0 to 24h, followed by measuring the protein levels of *mdig*, NRP1 and NRP2. In agreement with our previous findings 20, 21, we again found that As^3+^ is highly capable of inducing *mdig*, as well as NRP1 and NRP2 (Fig. 3B, left panels). A substantial reduction of NRP1 and NRP2 proteins were observed in the *mdig* KO cells relative to the WT cells (Fig. 3B, right panels). The role of *mdig* on NRP1 was also confirmed in both MDA-MB-231 breast cancer cells and A549 lung cancer cells. Knockout of *mdig* enhanced enrichment of H3K9me3 in the upstream of NRP1 gene, and H4K20me3, another repressive histone methylation marker, in the gene body and upstream of the NRP1 gene in MDA-MB-231 cells (sFig. 1), which correlated to a 2.5-fold decrease of the NRP1 protein in the KO cells as determined by quantitative proteomics (sFig.2). In A549 cells, knockout of *mdig* resulted in an enhancement of H4K20me3 in the down-stream of the NRP1 gene (sFig. 3). The *mdig* KO cells also exhibited reduced expression of AGTR1 (AT1), DPP4 and TMPRSS15 due to intensified enrichment of H3K9me3 on these genes (Fig. 3C). In human kidney cell line HK-2, AGTR1 is shown to be responsible in mediating endocytosis of SARS-CoV-2 in the presence of soluble ACE2 22. DPP4 serves as a receptor for MERS-CoV and possibly for SARS-CoV-2 too 23. Inhibitors of DPP4 conferred a 7-fold lower risk of COVID-19 mortality among patients with metabolic diseases 24. TMPRSS15 shares high homology in the serine protease domains of TMPRSS2, thus, may participate in the cleavage of the S protein of SARS-CoV-2 during infection. Both RAB6B and IQGAP2 are important regulators for lysosome-phagosome tethering and endocytic vesicle trafficking or fusion 25. Thus, the enhanced enrichment of H3K9me3 and/or H3K27me3 on and down-regulation of these genes in *mdig* KO cells suggest that *mdig* may facilitate SARS-CoV-2 infection through antagonizing the repressive histone methylation markers H3K9me3 and H3K27me3, leading to an upregulated expression of these genes. This notion is further supported by the decreased expression of the cathepsin (CTS) family members due to enhanced enrichment of H3K9me3 on these genes in *mdig* KO cells (Fig. 3D). Knockout of *mdig* caused strong enrichments of H3K27me3 and H3K9me3 in the up-stream of CTSD gene, which is consistent to the diminished CTSD protein in *mdig* KO cells as shown in Fig. 1A. Increased level of H3K9me3 was also seen on the gene bodies of CTSE, CTSL1, CTSL3P, and CTSL1P8 in *mdig* KO cells (Fig. 3D). Results from RNA-seq showed that except CTSF, the expression levels of all other members of the CTS family are significantly declined following *mdig* knockout (Fig. 3D, right panel). It had been well-documented that CTS members are the dominating proteases for further degradation of SARS-CoV-2 S proteins in lysosomes 26, 27, which is essential for the release of the viral RNA genome into the cytoplasm.

### The expression of the major glycosylation pathway genes is *mdig* dependent

**Figure 4:**
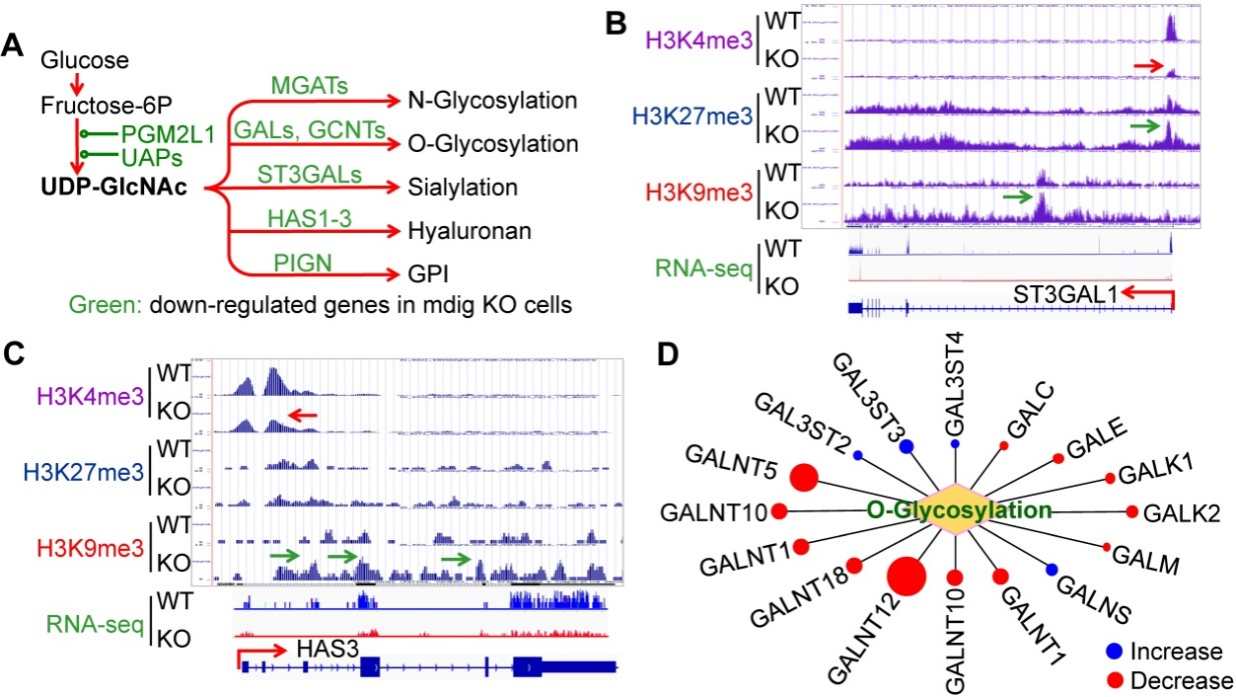
** Depletion of *mdig* decreased expression of the genes in protein glycosylation. A.** Diagram shows glucose metabolism and hexosamine biosynthetic pathway linked to glycan metabolism and glycosylation. The down-regulated genes in *mdig* KO cells are marked in green. **B and C.** Screenshot of ChIP-seq and RNA-seq for ST3GAL1 that catalyzes sialylation and HAS3 that catalyzes the generation of hyaluronan, respectively. Knockout of *mdig* enriched H3K9me3 and decreased H3K4me3, leading to the diminishment of the ST3GAL1 and HAS3 mRNA in RNA-seq. **D.** Down-regulation of O-glycosylation pathway genes in *mdig* KO cells. Red circles denote decreased, while blue circles denote an increased expression of the genes in *mdig* KO cells. The sizes of circles indicate the degree of increase or decrease of these gene expressions in *mdig* KO cells.

Glycosylation creates great structural and functional diversity of the target proteins. During SARS-CoV-2 infection, glycosylation of the viral proteins is essential for the assembly of the virion and is able to shield the virus from the host immune response 28. One of the major glycosylation pathways is the hexosamine biosynthetic pathway (HBP) from glycolytic metabolism of the glucose, which generates uridine diphosphate N-acetylglucosamine (UDP-GlcNAc), the founding molecule for N- and O-glycosylation, sialylation, formation of hyaluronan, and biosynthesis of glycosylphosphatidylinositol (GPI) (Fig. 4A). In the *mdig* KO cells, we observed a strong enrichment of the repressive histone marker, H3K9me3, on the genes in this pathway, such as ST3GAL1 (Fig. 4B) and HAS3 (Fig. 4C), along with a diminishment of the active methylation marker H3K4me3. RNA-seq confirmed a decreased expression of these two genes and other genes as indicated in Fig. 4A. Interestingly, most of the GalNAc kinases (GALs) that exhibited increased enrichment of H3K9me3 or H3K27me3 in ChIP-seq, are down-regulated as determined by RNA-seq in the *mdig* KO cells (Fig. 4D). Accordingly, we believe that in the normal cells, induction of *mdig* will enforce the overall glycosylation for both viral and host cell proteins.

### Upregulation of NRP1, NRP2 and glycosylation pathway genes in As^3+^-transformed cells

**Figure 5:**
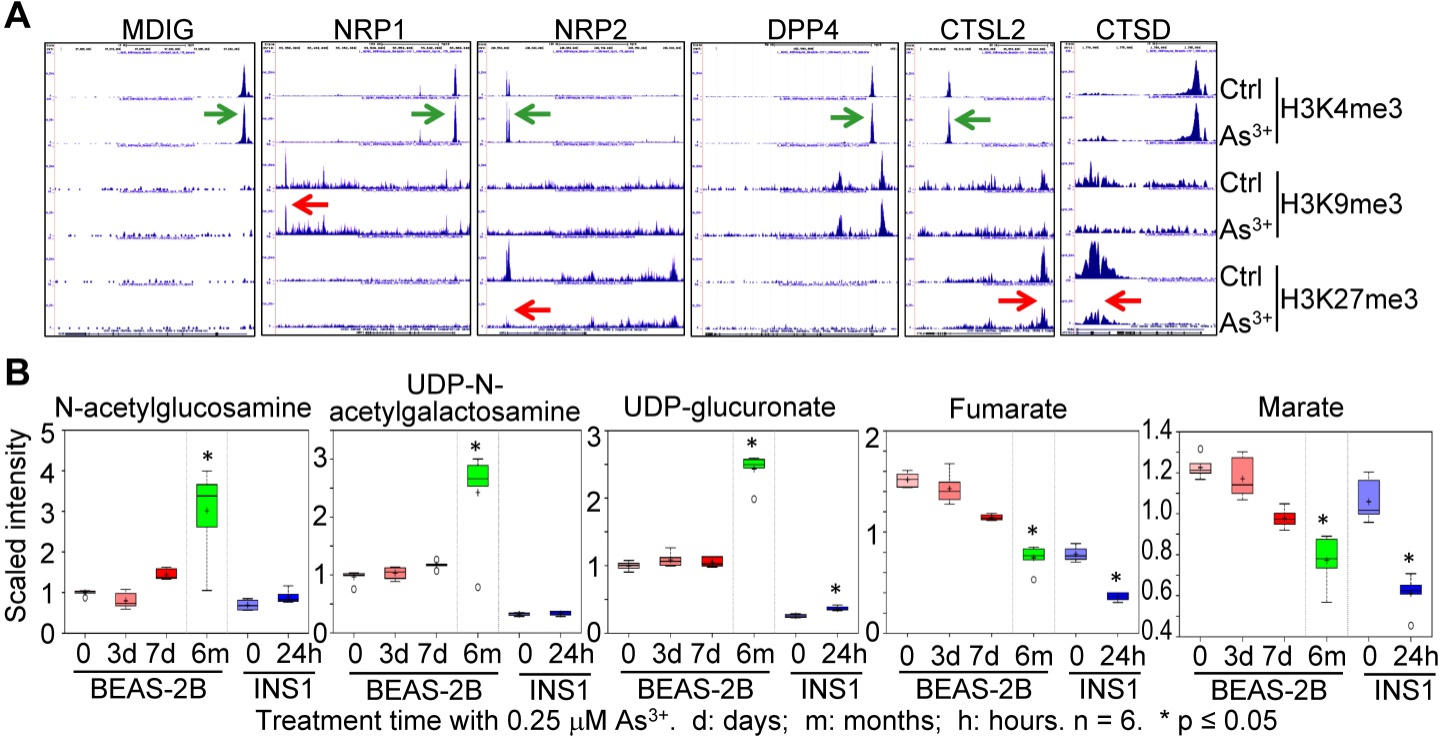
** Environmental factor As^3+^ induces expression of genes for SARS-CoV-2 infection and increases metabolites in protein glycosylation. A.** Screenshot of ChIP-seq of the indicated genes in control and As^3+^-transformed cells. **B.** Metabolomics analysis of the indicated precursor metabolites for protein glycosylation and metabolites in mitochondrial citric acid cycle in BEAS-2B and INS1 cells treated with 0.25 µM As^3+^ for the indicated times. d: days; m: months; h: hours.

As^3+^ is one of the most common environmental carcinogens linked to lung cancer and other diseases 29. Previous studies indicated induction of *mdig* by As^3+^ in both BEAS-2B cells and A549 cells 20, 21. Because of the *mdig*-dependent expression of NRP1, NRP2 (Fig. 3), and genes in glycosylation (Fig. 4), we examined the enrichment levels of active methylation, H3K4me3, and the repressive methylation, H3K9me3 and H3K27me3 on the genes associated with SARS-CoV-2 infection and the pathogenesis of COVD-19 in the cells that were consecutively treated with 0.25 μM As^3+^ for six months to induce transformation 30, 31. An enhancement of H3K4me3 was noted on the genes of *mdig*, NRP1, NRP2, DPP4, and CTSL2 in the As^3+^-transformed cells (Fig. 5A). However, the levels of H3K9me3 on NRP1, and H3K27me3 on NRP2, CTSL2, and CTSD were decreased substantially. These results indicated an active transcription and expression of these genes in the As^3+^-transformed cells, which provides strong evidence linking environmental factors to the infection of SARS-CoV-2.

The As^3+^-transformed cells showed a metabolic shift from mitochondrial citric acid cycle to glycolysis, the latter generates precursor metabolites for protein glycosylation 30. Treatment of the BEAS-2B cells with 0.25 µM As^3+^ for 3 to 7 days or until 6 months, and the rat insulinoma cells INS1 with 0.25 µM As^3+^ for 24 h, induced generation of glycosylation precursors N-acetylglucosamine, UDP-N-acetylgalactosamine and UDP-glucuronate in metabolomics analysis (Fig. 5B). In contrast, a time-dependent decrease of mitochondrial citric acid cycle metabolites, fumarate and marate, was observed in both BEAS-2B cells and INS1 cells treated with 0.25 μM As^3+^ for the indicated times. These data, thus, provided complementary evidence supporting the connections between environmental exposure and the pathogenesis of COVID-19.

### *Mdig* is a master regulator of inflammation

**Figure 6:**
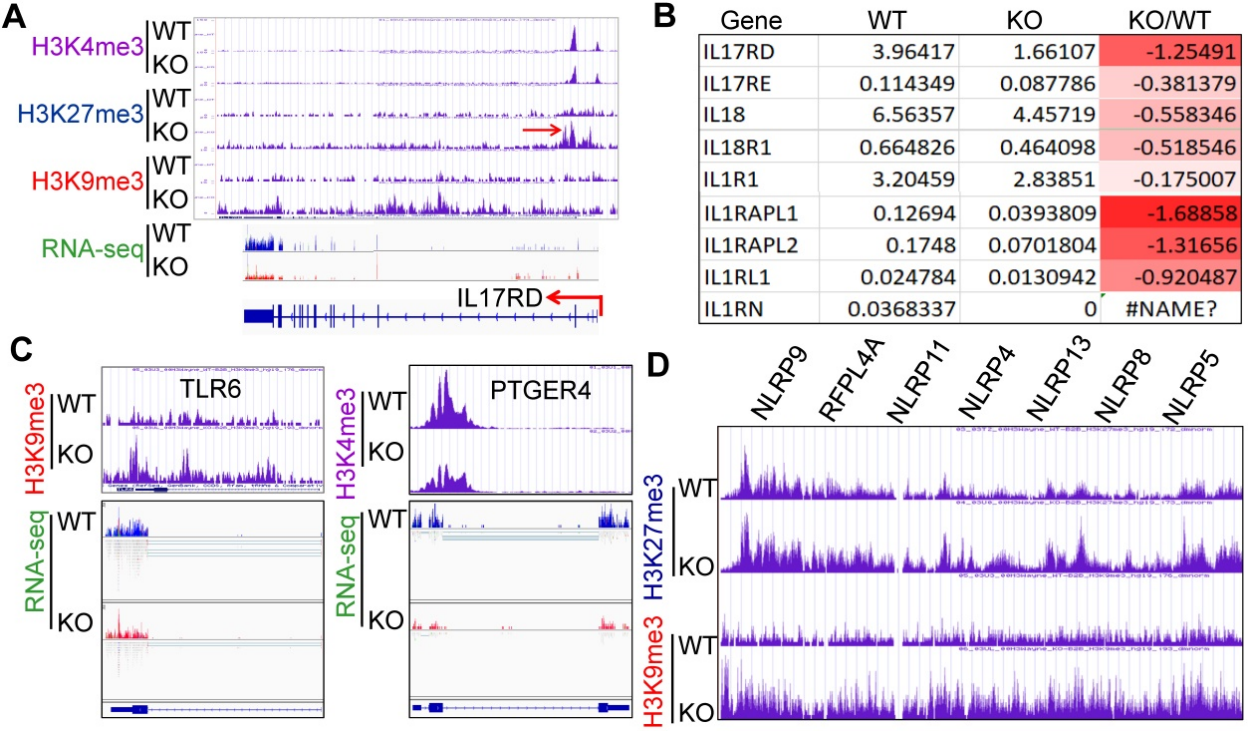
** Regulatory role of *mdig* on inflammation. A.** ChIP-seq and RNA-seq of IL17RD in both WT and *mdig* KO cells. Red arrows point to the enhanced peaks of H3K27me3 and H3K9me3 on the IL17RD gene in *mdig* KO cells. **B.** RNA-seq shows down-regulation of these indicated inflammatory cytokine receptors or their regulatory protein in *mdig* KO cells. **C.** ChIP-seq shows increased enrichment of H3K9me3 on TLR6 gene and decreased enrichment of H3K4me3 on PTGER4 gene in *mdig* KO cells, which correlated to the diminished expression of these genes in RNA-seq (bottom panels). **D.** Enrichment of H3K27me3 and H3K9me3 on the NLRP gene loci that encode the key components of inflammasomes.

Inflammatory cytokine storm is the most important mortality factor of COVID-19 patients. Our recently published data demonstrated that *mdig* controls the expression of genes involved in inflammation, including TGFβ signaling, collagens and cell adhesion molecules 17, 18. In addition, heterozygotic knockout of *mdig* in mice ameliorated silica-induced lung fibrosis along with a significant decrease of Th17 cell and macrophage infiltration into the lung interstitium 18, 19. Re-analyzing of the ChIP-seq and RNA-seq data from the WT and *mdig* KO cells further unraveled that *mdig* regulates a wide spectrum of inflammatory genes, such as several receptor genes for IL17 and IL1 (Figs. 6A and 6B). Knockout of *mdig* caused an elevated enrichment of H3K27me3 and H3K9me3, both are repressive markers for gene transcription, on IL17RD. Meanwhile, the expression of some TLR and NLRP members involved in inflammasome-mediated inflammation are also compromised in the *mdig* KO cells (Figs. 6C and 6D). On the genes encoding prostaglandin E2 receptors 2, 3 and 4, although there was no significant gain of H3K9me3 and H3K27me3, a notable reduction of H3K4me3, an active marker for gene transcription, on these genes was observed in the KO cells (Fig. 6C).

### Elevated expression of *mdig* in pulmonary fibrosis

**Figure 7:**
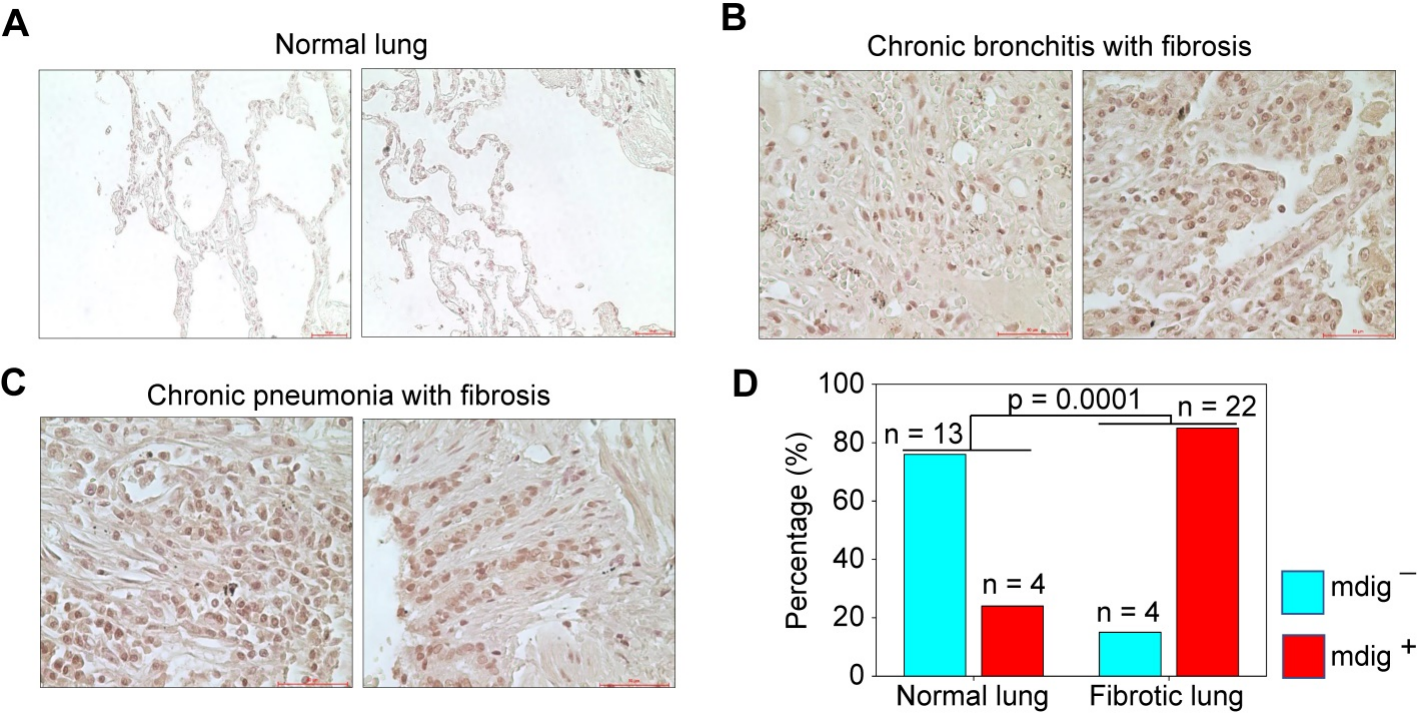
** Immunohistochemical staining of *mdig* in normal and fibrotic human lung tissues. A.** Staining of *mdig* in representative normal human lung tissues. A total of 17 normal lung tissue samples were investigated. **B.** Staining of *mdig* in fibrotic lung tissues from patients with chronic bronchitis. **C.** Staining of *mdig* in fibrotic lung tissues from patients with chronic pneumonia. **D.** Summary of *mdig* positive rates in normal and fibrotic human lung tissues.

Pulmonary fibrosis is one of the major contributing factors to the acute respiratory distress syndrome (ARDS) of the COVID-19 patients, which is also a common sequela in some COVID-19 survivors 32, 33. Many fibrotic damages are irreversible and may cause lifelong functional impairment of the lung. The pro-fibrotic role of *mdig* was first demonstrated in silica-induced lung fibrosis in mice 17-19. It is unknown whether *mdig* is also important in the development of lung fibrosis in humans. To answer this question, we screened *mdig* expression through immunohistochemistry in tissue microarrays containing 17 normal human lung tissues and 26 cases of fibrotic lung tissues collected from patients with cancer, chronic bronchitis or chronic pneumonia. In agreement with earlier reports that *mdig* was barely detectable in normal lung tissue 34, 35, in this analysis, only 24% of normal lung tissues showed some faint staining of *mdig*. In contrast, the majority of fibrotic lung tissues, 85%, exhibited a strong signal of *mdig* expression (Fig. 7). Although we cannot distinguish the question of whether *mdig* promoted fibrosis or fibrosis caused a higher expression of *mdig* in human lung tissues, based on previous mouse model with *mdig* knockout and the fact that *mdig* is a master regulator of inflammation 17-19, we believe that *mdig* is a critical driving factor for the development of lung fibrosis associated with some disease conditions, such as COVID-19, chronic bronchitis, pneumonia, and cancer.

### Contribution of *mdig* to the COVID-19 severity associated with the Neanderthal variants

**Figure 8:**
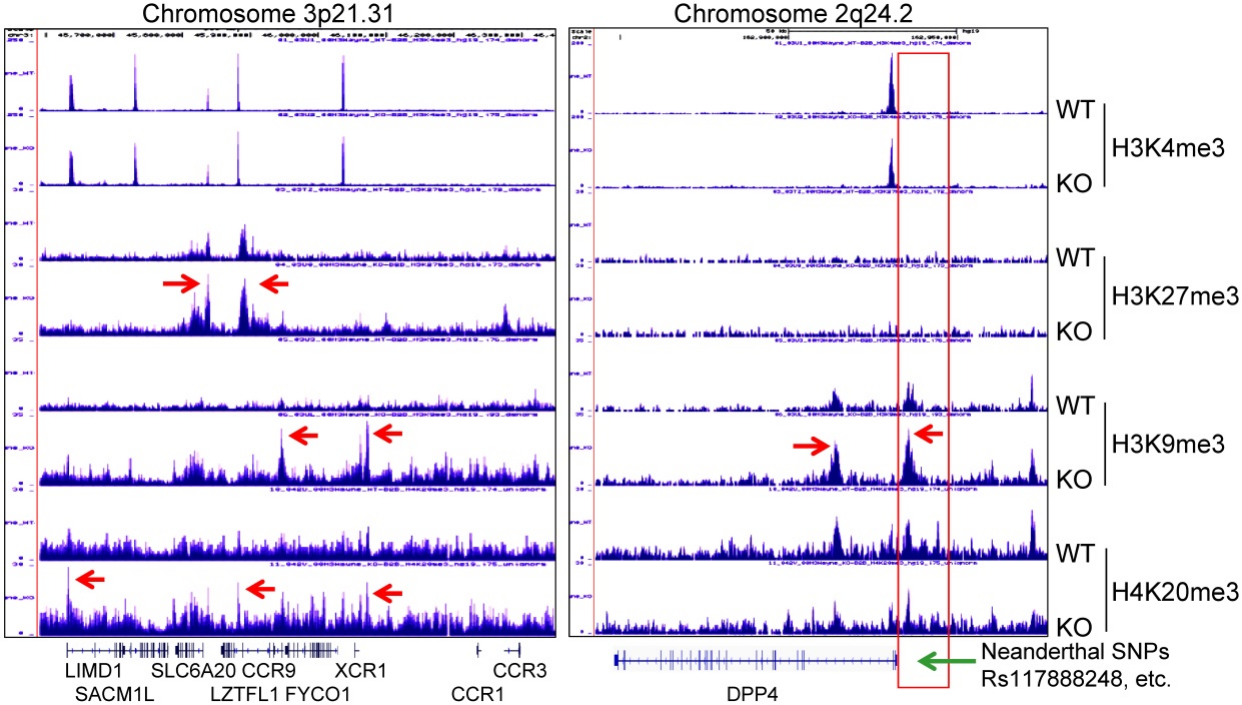
** Knockout of *mdig* enhances enrichment of the repressive histone trimethylation markers on the Neanderthal variants that increase the risk of severe COVID-19.** Left panel shows Neanderthal haplotype region in Chromosome 3p21.31. Red arrows point to the elevated peaks of H3K9me3, H3K27me3 and H4K20me3 in the *mdig* KO cells. Right panel shows increased level of H3K9me3 in the gene body and promoter of DPP4 as indicated by red arrows. Red box denotes the promoter region of DPP4 encompassing all Neanderthal single nucleotide polymorphisms (SNPs) that increase the risk of severe COVID-19, including rs117888248, rs79624636, rs116906287, rs118098838, rs76135328, rs117460501, rs57355997, rs188765526, and others.

A recent genome-wide association study of COVID-19 revealed connections between the disease severity and two genetic regions in chromosomes 3p21.31 and 9q34.2 36. Follow-up study suggested that the specific genetic variants derived from a Neanderthal heritage in six genes located in Chromosome 3p21.31 are the major risk factors for severe COVID-19 37. Our ChIP-seq data demonstrated an increased enrichment of H3K9me3 and H4K20me3 in this region in the *mdig* KO cells relative to the WT cells, indicating regulatory role of *mdig* on these genes in this region (Fig. 8, left). Although it is unclear on the functions of some of these genes in this region during SARS-Cov-2 infection, an earlier report suggested direct interaction of SLC6A20 and ACE2 for amino acid transportation 38, indicating possible involvement of these genes in regulating S protein binding of SARS-Cov-2 to ACE2. Most recent study also unraveled that another Neanderthal variants at chromosome 2q24.2 on the proximal promoter region of DPP4 can double or quadruple the risk of becoming critically ill with COVID-19, such as hospitalization and requiring mechanical ventilation 39. In *mdig* KO cells, we noted strong enrichment of H3K9me3 and H4K20me3 in the upstream of the DPP4 gene encompassing all of these known Neanderthal variants (Fig. 8, right), which correlated a 2-fold decrease of DPP4 transcription in KO cells as determined by RNA-seq (Fig. 3C). Intriguingly, knockout of *mdig* has no effect on the enrichment of repressive histone trimethylation markers, H3K9me3, H3K27me3 or H4K20me3, on the protective Neanderthal haplotypes in chromosome 12 that reduce the risk of becoming severely ill upon SARS-CoV-2 infection 40, nor on the transcription of these genes, including OAS1, OAS2 and OAS3, in this region that target RNA virus, (data not shown).

## Discussion

**Figure 9:**
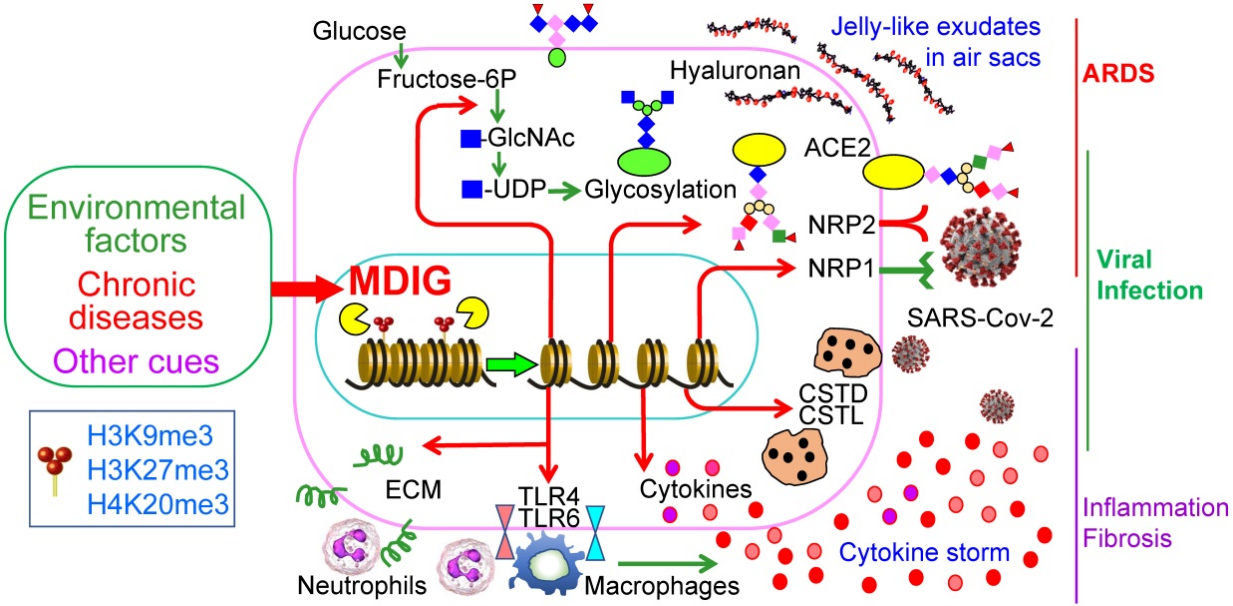
Schematic diagram shows regulatory roles of environmental factor-induced *mdig* on the infectivity of SARS-CoV-2 and the pathogenesis of COVID-19.

The COVID-19 pandemic due to SARS-CoV-2 infection has many destructive effects on public health and social-economics on a global scale. Despite a large amount of molecular detail is documented about the virology of SARS-CoV-2 and the pathogenesis of COVID-19, little is currently known about why some COVID-19 patients showed mild or no symptoms but others, even who are at young ages and have no pre-existing disease conditions, have severe or fatal outcomes. A number of studies had demonstrated that several environmental factors, including air pollution, exposure to perfluorinated alkylates (PFASs), temperature, humidity, wind speed, etc. had a significant influence on the mortality of COVID-19 14-16, 41. However, to the best of our knowledge, no detailed studies have investigated how these suspected environmental factors intensify the severity or mortality of COVID-19. In this report, we provided evidence suggesting that *mdig*, a potential oncogenic gene induced by several common environmental hazards 17, 42, may augment the infectivity of SARS-CoV-2 and the severity of COVID-19 through upregulating S protein receptors NRP1 and NRP2, several proteases and the genes in the pathways of glycan metabolism and inflammation (Fig. 9).

It has been a general assumption that ACE2 is abundantly expressed in bronchial epithelial cells and other lung cells, and serves as the main receptor for the S protein of SARS-CoV-2 43, 44. It was beyond our expectation that ACE2 was barely detected by RNA-seq in both WT and *mdig* KO BEAS-2B cells (Fig. 3C), a cell line derived from non-cancerous human bronchial epithelial cells. Although we cannot rule out the possibility of genetic or epigenetic variations during the establishment and passage of the cell lines, there is evidence showing very low protein level of ACE2 in respiratory epithelial cells 45. Meanwhile, proteomic analysis of autopsy samples from COVID-19 patients also revealed that ACE2 was barely detected in the lung 46. Interestingly, a recent retrospective analysis indicated that compared to children, the expression level of ACE2 in lower pulmonary tract is much decreased in older COVID-19 patients who showed severe symptoms and poor prognosis 47. Among pediatric COVID-19 patients, although it was inconclusive, histological examination of lung biopsy samples suggested that the difference in disease severity between infants and older children appears to be independent on the expression and cell type distribution of ACE2 44. Accordingly, it is very likely that NRP1 and NRP2 may be critically required for efficient viral entry into the host cells in the lung. The dependence of *mdig* on the expression of NRP1 and NRP2 was also observed in two additional cell lines, MDA-MB-231 (sFig.1 and sFig. 2) and A549 (sFig. 3). Thus, it is plausible to speculate that *mdig* dependent expression of NRP1 and NRP2 can enhance the infectivity of SARS-CoV-2, leading to the worsening effect of environmental exposure on the severity of COVID-19.

Sequential cleavage of the S protein at S1-S2 boundary and S2' site by host proteases is a prerequired step for conformational changes and membrane fusion of the S protein, followed by viral internalization and intracellular trafficking within endosomes and lysosomes. Inside the lysosomes, the S and other proteins on the surface of SARS-CoV-2 will be subjected to additional cleavages by cathepsins, primarily by CTSL and CTSB. A number of studies concluded that inhibitors that target lysosome or the lysosomal proteases, cathepsins, can prevent the release of the viral RNA genome into the host cytoplasm and viral entry into the target cells 48. Decreased expression of CTSB, CTSD, CTSE, and CTSL members in the *mdig* KO cells suggests that *mdig* bolsters the expression of proteases required for additional cleavage of the S protein, which provides a permissive environment for SARS-CoV-2 propagation. Indeed, most recent proteomic analysis of COVID-19 autopsies suggested an overall upregulation of the cathepsin family members, including CTSB, CTSD, CTSE, CTSH, CTSK, CTSS, and CTSZ, in the lung of the COVID-19 patients 49.

Both S protein of SARS-CoV-2 and ACE2 are highly glycosylated 50. Certainly, glycosylation changes the antigenicity of S protein and its affinity towards cellular receptors. Meanwhile, membrane fusion of SARS-CoV-2 with the host cells is strengthened by some types of glycosylation, such as sialylation, of the cell surface proteins, 51. More importantly, in addition to intensify the infectivity of the SARS-CoV-2, amplified glycosylation causes excessive generation of hyaluronan that forms liquid jelly-like structure in both alveolar space and the interstitial area, which is fatal for COVID-19 patients 52. Inflammatory cytokine storm is one of the contributing factors for the excessive generation of hyaluronan based on the fact that these cytokines are strong inducers of hyaluronan synthase-2 (HAS2) 53. Our previous 17-19 and the current report unfolded that *mdig* is largely responsible for the expression of inflammatory cytokines and the key component of the inflammasome, as well as most of the genes in glycan metabolism, such as Has1, Has2 and Has3 for hyaluronan generation, and GALs for O-glycosylation (Fig. 4). These effects of *mdig* are also attributable to the pulmonary fibrosis among some COVID-19 survivals 32, 33. The elevated level of *mdig* in fibrotic human lung tissues (Fig. 7) supports this notion. Thus, over-expression of *mdig*, either under some chronic disease conditions or environmental exposure, possibly indicates the pathogenetic mechanism behind the severity or mortality of COVID-19.

The data from this report yield a conceivable explanation on how environmental exposure exacerbates the severity and/or mortality of COVID-19. However, additional studies are much needed to overcome major limitations due to lack of patient data and to determine whether *mdig* plays critical roles on the infection of SARS-CoV-2 and the severity of COVID-19 among patients who have history of environment exposure. A future population-based meta-analysis as well as animal models will be valuable to provide direct evidence indicating *mdig*-dependent expression of NRP1 or NRP2 that enhances SARS-CoV-2 infection of the cells that have lower level of ACE2 expression. The contribution of *mdig*-dependent expression of NRP1, NRP2 and genes in inflammation and glycosylation, to the pathogenesis of COVID-19 does not challenge the fact that ACE2 is the main receptor of SARS-CoV-2. A more complete understanding of *mdig* and its regulatory pathways will catalyze effective management strategies that ameliorate the infectivity of SARS-CoV-2 and improve the outcomes of the COVID-19 patients.

## Materials and methods

### Cell culture

The human bronchial epithelial BEAS-2B cells were purchased from America Type Culture Collection (ATCC). The arsenic-transformed cells were described previously 30. The rat inslinoma cell line INS1 was a gift from Dr. Anjaneyulu Kowluru, Professor of Pharmaceutical Sciences, Wayne State University. All cells were cultured in DMEM-high glucose medium (Sigma, cat.no. D5796) supplemented with 5% FBS, 1% penicillin-streptomycin (Gibco, cat.no. 15140122), and 1% L-Glutamine (Gibco, cat.no. 25030164). Cells were maintained in a humidified incubator at 37 °C with 5% CO_2_. In some experiments, the cells were treated with 0.25 to 2 μM arsenic as reported previously 30.

### Establishment of *mdig* knockout (KO) cells by CRISPR-Cas9

For generation of CRISPR-Cas9 plasmids, sgRNA (5'-AATGTGTACATAACTCCCGC-3') was selected and cloned into pSpCas9(BB)-2A-Blast vector as described 18. BEAS-2B cells (2.5 x 10^5^/well in 6-well plate) were transfected with CRISPR-Cas9 plasmids by Lipofectamine 2000 (Thermo Fisher Scientific) according to the manufacturer's protocol. The transfected cells were selected with 2 µg/ml Blasticidin (Thermo Fisher Scientific) until single colonies were formed. WT and *mdig* KO colonies were validated by measuring MDIG protein expression.

### SARS-CoV2-Spike transfection

WT and *mdig* KO cells (3 × 10^5^/well in 6-well plate) were seeded, and grown to 70% confluence. SARS-CoV2-Spike plasmids (Addgene, cat.no. 145032) were transfected into cells using Lipofectamine 2000 (Thermo Fisher Scientific) according to the manufacturer's protocol. Twenty-four hours after transfection, cells were subjected to western blotting.

### Western blotting

Cells were lysed in 1 x RIPA buffer (Cell Signaling), supplemented with 1mM PMSF and protease inhibitor cocktail (Thermo Fisher Scientific). Protein concentrations were determined by Pierce BCA Protein Assay Kit (Thermo Fisher Scientific). Whole cell lysates (30 µg) were separated in sodium dodecyl sulfate-polyacrylamide gel electrophoresis (SDS-PAGE), and then transferred to Polyvinylidene fluoride (PVDF) membranes (Millipore). The membranes were blocked with 5% nonfat dry milk and then probed with primary antibodies at 4 °C overnight. The primary antibodies include anti-*mdig* (Invitrogen cat.no. 39-7300), anti-SARS-CoV-2-Spike, anti-NRP1 and NRP2 (Abcam, ab272504), anti-Furin (Abcam, ab183495), anti-TMPRSS2 (Proteintech, cat. no. 14437-1-AP), anti-Cathepsin D (Cell signaling, cat. no. 2284S), and anti-GAPDH (Cell signaling, cat. no. 2118S). After washing with TBST (3 × 10 min), the membranes were incubated with horseradish peroxidase-coupled goat anti-rabbit or -mouse IgG at room temperature for 1h and washed with TBST (3 × 10 min). Chemiluminescent Substrate (Thermo Fisher Scientific) was used to detect immunoblotting signals. Data are representatives of at least three independent experiments. Semi-quantification of some Western blotting data was performed through density scanning using ImageJ software and calibrated by the density of GAPDH or histone H3.

### Chromatin immunoprecipitation-sequencing (ChIP-seq)

Two sets of ten million WT or KO BEAS-2B cells were fixed and subjected to ChIP with ChIP-grade antibodies against H3K9me3, H3K27me3, H3K4me3, and H4K20me3 (Active Motif, CA). ChIP, input/control DNA libraries, NGS sequencing, and data analysis were performed as we described previously 18. Enrichment differences between WT and KO cells for these histone methylation markers on individual genes as indicated in figures were determined by randomly selecting three highest peaks at the same genetic location on UCSC Genome Browser between WT and KO cells to calculate the means and p values through *t*-test.

### RNA sequencing (RNA-seq)

Two million WT or KO BEAS-2B cells were collected for total RNA isolation, libraries preparation and NGS sequencing. The procedures and data analysis were followed as we previously reported 18.

### Metabolomics

Untargeted global metabolomics was performed as previously described 30. Briefly, six replicates of each control or cells treated with 0.25 μM As^3+^ at different time-point were prepared for metabolomics analysis through Ultrahigh Performance Liquid Chromatography-Tandem Mass Spectroscopy (UPLC-MS/MS). Raw data was extracted, peak-identified and QC processed. Compounds were identified by comparison to library entries of purified standards or recurrent unknown entities. Multiple statistical analyses, including Welch's two-sample *t*-test, Matched pairs *t*-test, One-way and Two-way ANOVA, Hotelling's T^2^ test, etc., were applied at the different stages of data processing. The data were first sorted by the p-value with a cutoff of p < 0.05 and then subjected to q-value calculation. High statistical significance was given to data with q < 0.05 along with a false discovery rate (FDR) 2.5%.

### Immunohistochemistry

Pulmonary interstitial fibrosis tissue microarray LC561 was purchased from the USBiomax, Inc. The demographic information of the patients is available from USBiomax website. Tissue microarray slides were processed for immunohistochemical staining for *mdig* as described previously 34. Briefly, paraffin-embedded tissue sections were deparaffinized with xylene and hydrated in a series of alcohol gradients. To quench endogenous peroxidase activity, slides were incubated with 1.5 to 3% H_2_O_2_ in PBS for 20 min at room temperature. Heat-mediated antigen retrieval was performed by boiling tissue sections in citrate buffer with pH 6.0 for 20 min in a microwave. To block nonspecific binding of immunoglobulin, slides were incubated with a solution containing 5% goat serum, 0.2% Triton X-100 in PBS for 2 h at room temperature, followed by incubation with primary antibodies against *mdig* (mouse anti-MINA, Invitrogen with 1:50 dilution) overnight at 4 °C. Goat anti-mouse biotinylated secondary antibodies were subsequently applied at 1:200 dilution and incubated for 2 h at room temperature. Slides were then incubated with ABC reagent (Vectastatin Elite ABC kit) for 45 min at room temperature, and the chromogen was developed with diaminobenzidine (DAB). Slides were counterstained with hematoxylin (Sigma-Aldrich, St. Louis, MO) and mounted with Entellan® (Electron Microscopy Sciences, Hatfield, PA). All incubation steps were carried out in a humidified chamber, and all washing steps were performed with 1 × PBS. Images were captured under the bright field of a Nikon Eclipse Ti-S Inverted microscope (Mager Scientific, Dexter MI, USA) and analyzed using Nikon's NIS Elements BR 3.2 software.

### Statistics and data analysis

Expression profiles of GSE149312, GSE150392, and GSE147507 were obtained from the public NCBI-GEO database. Gene set enrichment analyses comparing RNA-seq analyses were performed using the online public tool Enrichr (http://amp.pharm.mssm.edu/Enrichr/). Pathway analyses were performed by Reactome Knowledgebase (www.reactome.org). For Western blotting, at least three independent experiments were performed. Statistical significance for some quantitative data was determined by unpaired Student's *t*-test.

## Supplementary Material

Supplementary figures.
