# Supplementary material for: Environmentally-induced mdig contributes to the severity of COVID-19 through fostering expression of SARS-CoV-2 receptor NRPs and glycan metabolism
Source: Theranostics. 2021 Jul 6;11(16):7970–83. doi: 10.7150/thno.62138 (PMC8315075; doi:10.7150/thno.62138)
Supplement: Supplementary file 1 — Supplementary figures. [file thnov11p7970s1.pdf]

Supplement figure legends

sFig. 1. ChIP-seq of NRP1 gene in WT and *mdig* KO MDA-MB-231 breast cancer cell line. Red arrows point to the enhanced enrichment of H3K9me3 and H4K20me3 in *mdig* KO cells.

sFig. 2. Quantitative proteomics analysis for the protein levels of NRP1 in 5 WT (red) and 11 *mdig* KO (green) MDA-MB-231 cell clones. Duplicated samples for each clone were subjected to quantitative proteomics analysis. One sample in each group was discarded in this analysis due to poor quality from sample preparation and processing.

sFig. 3. ChIP-seq of NRP1 gene in WT and *mdig* KO A549 cells. Red arrow points to the elevated enrichment of H4K20me3 on the NRP1 gene in *mdig* KO A549 cells.

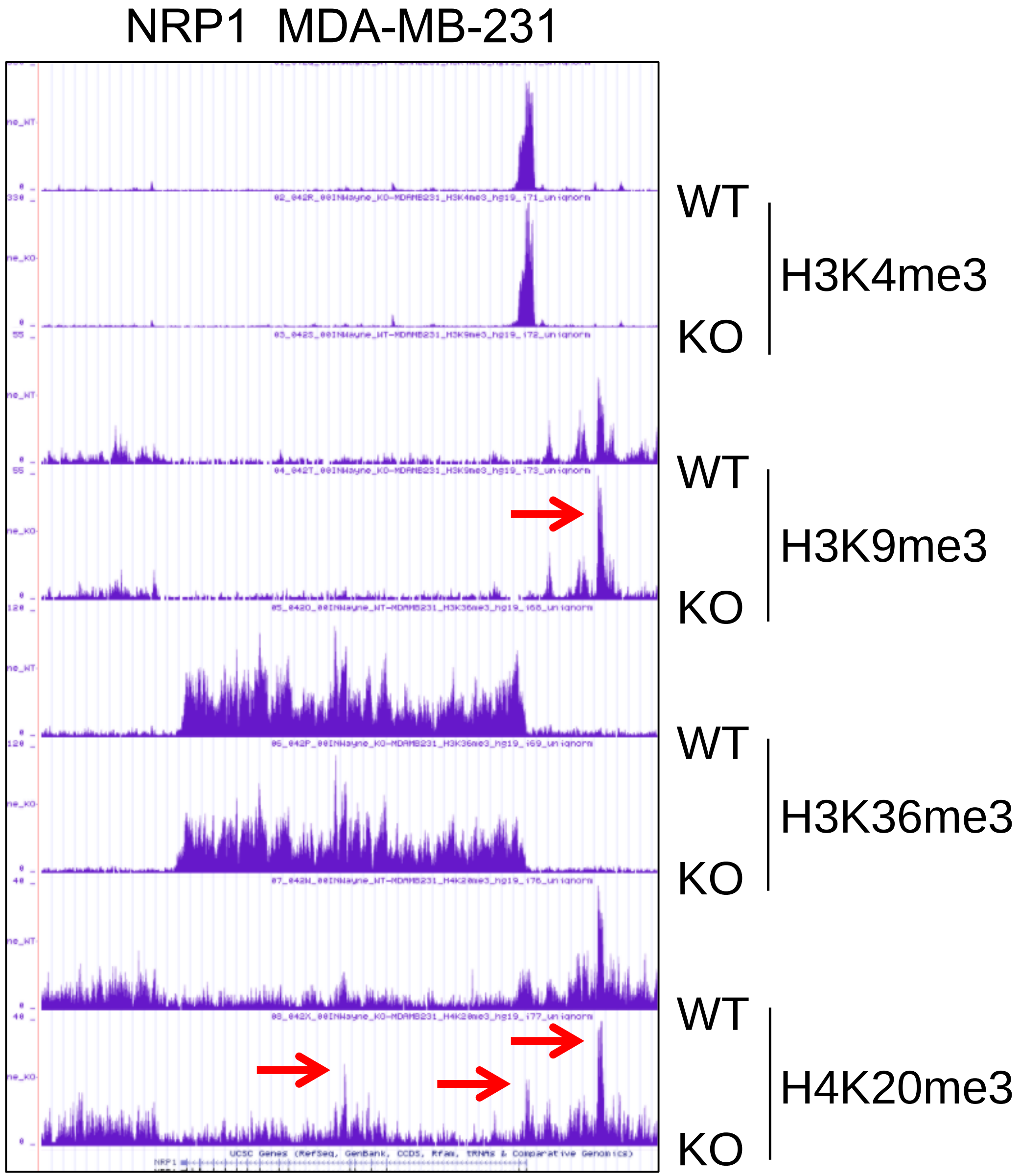
NRP1 MDA-MB-231
WT
KO
H3K4me3
WT
KO
H3K9me3
WT
KO
H3K36me3
WT
KO
H4K20me3

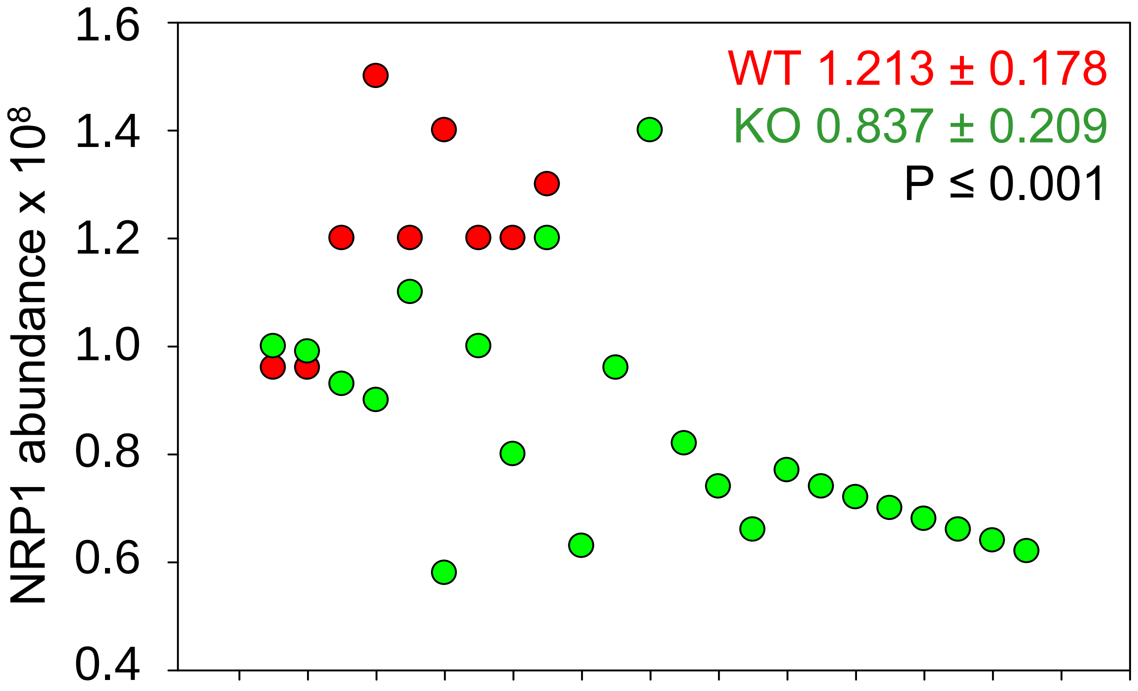

WT 1.213 ± 0.178
KO 0.837 ± 0.209
P ≤ 0.001
NRP1 abundance x 10^8
1.6
1.4
1.2
1.0
0.8
0.6
0.4

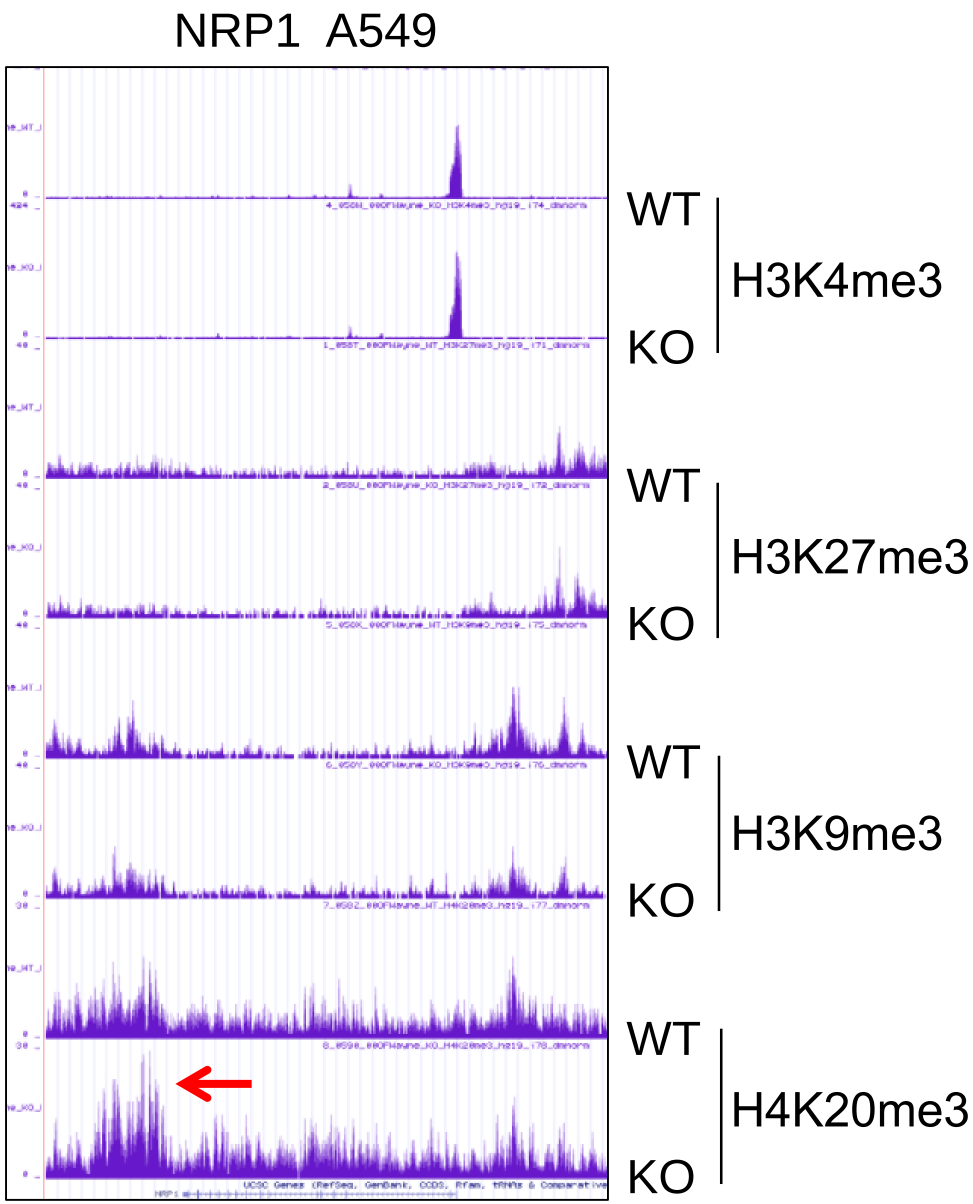
NRP1 A549
WT
KO
H3K4me3
WT
KO
H3K27me3
WT
KO
H3K9me3
WT
KO
H4K20me3
